# Weed Classification for Site-Specific Weed Management Using an Automated Stereo Computer-Vision Machine-Learning System in Rice Fields

**DOI:** 10.3390/plants9050559

**Published:** 2020-04-27

**Authors:** Mojtaba Dadashzadeh, Yousef Abbaspour-Gilandeh, Tarahom Mesri-Gundoshmian, Sajad Sabzi, José Luis Hernández-Hernández, Mario Hernández-Hernández, Juan Ignacio Arribas

**Affiliations:** 1 Department of Biosystems Engineering, College of Agriculture and Natural Resources, University of Mohaghegh Ardabili, Ardabil 56199-11367, Iran; m.dadashzadeh@uma.ac.ir (M.D.); mesrigtm@uma.ac.ir (T.M.-G.); s.sabzi@uma.ac.ir (S.S.); 2 Division of Research and Graduate Studies, TecNM/Technological Institute of Chilpancingo, Chilpancingo 39070, Mexico; joseluis.hernandez@itchilpancingo.edu.mx; 3 Faculty of Engineering, Autonomous University of Guerrero, Chilpancingo 39070, Mexico; mhernandezh@uagro.mx; 4 Department of Teoría de la Señal y Comunicaciones, University of Valladolid, 47011 Valladolid, Spain; 5 Castilla-León Neuroscience Institute, University of Salamanca, 37007 Salamanca, Spain

**Keywords:** sustainable agriculture, site-specific management, eco-friendly technique, weed, rice field, metaheuristic algorithm

## Abstract

Site-specific weed management and selective application of herbicides as eco-friendly techniques are still challenging tasks to perform, especially for densely cultivated crops, such as rice. This study is aimed at developing a stereo vision system for distinguishing between rice plants and weeds and further discriminating two types of weeds in a rice field by using artificial neural networks (ANNs) and two metaheuristic algorithms. For this purpose, stereo videos were recorded across the rice field and different channels were extracted and decomposed into the constituent frames. Next, upon pre-processing and segmentation of the frames, green plants were extracted out of the background. For accurate discrimination of the rice and weeds, a total of 302 color, shape, and texture features were identified. Two metaheuristic algorithms, namely particle swarm optimization (PSO) and the bee algorithm (BA), were used to optimize the neural network for selecting the most effective features and classifying different types of weeds, respectively. Comparing the proposed classification method with the K-nearest neighbors (KNN) classifier, it was found that the proposed ANN-BA classifier reached accuracies of 88.74% and 87.96% for right and left channels, respectively, over the test set. Taking into account either the arithmetic or the geometric means as the basis, the accuracies were increased up to 92.02% and 90.7%, respectively, over the test set. On the other hand, the KNN suffered from more cases of misclassification, as compared to the proposed ANN-BA classifier, generating an overall accuracy of 76.62% and 85.59% for the classification of the right and left channel data, respectively, and 85.84% and 84.07% for the arithmetic and geometric mean values, respectively.

## 1. Introduction

Rice is considered a staple food for over half of the world’s population [1]. Weeds are among the most significant factors decreasing the yield of rice, incurring not only major economic costs, but also crop quality issues. Crops may be affected by weeds at any stage of growth. The weeds compete with crops in absorbing water, sunlight, and nutrition [2,3,4,5]. Early weed control can not only prevent the loss of crop yield by up to 34%, but can also lower the occurrence of pests and diseases [6,7]. In this case, chemical and non-chemical weed control techniques have been widely used across rice fields. As a non-chemical method, manual weeding is too tedious, costly, and time-consuming. An alternative non-chemical method is mechanical weed control. The machine vision technology provides a tool for real-time tracing of weeds by a robotic mechanical weeding system. Appropriate detection of the plant rows and discrimination of crops from weeds are the core challenges faced by such robots. Choi et al. (2015) proposed a new algorithm for guidance line extraction using morphological characteristics of the rice plant on images from the crop rows. The respective robot was equipped with screw-type wheels for weeding [8]. Nakai and Yamada (2014) developed a new robot for weed control in rice fields. The robot had a laser range finder and stereo camera for autonomous mobility and a robotic arm for weed control [9]. Identification of obstacles, rice plants, and weeds in different growth stages and without damaging the rice has been a challenging activity. Moreover, implementation of robotic weeding to traditional planting schemes and non-row cropping farms has been seen to require complex software and hardware technologies [10,11]. 

Based on the application of herbicides, the chemical method represents the traditional approach to weed removal and provides relative high weed control efficacy [5,12,13]. The efficacy of chemical weed control in terms of crop yield has been studied in many research works [14,15,16,17]. Apart from the advantages of using herbicides for weed control, such a control also suffers from drawbacks. Drawbacks are mainly due to the traditional spraying technology that leads to excessive use of herbicides and may end up with some serious problems, such as environmental pollution and ecological and safety issues [13]. This highlights the importance of exploring sustainable approaches to reducing the use of agrochemicals while retaining the efficacy. Weed detection technology for precision spraying is a suitable approach for optimizing herbicide use and reducing environmental impacts [18]. 

At present, development of computer vision and automatic expert systems has made it possible to distinguish between crops and weeds in an easy, yet fast fashion. Various ground-based weed detection techniques have been studied for selective herbicide application and site-specific weed management as eco-friendly techniques for reducing the consumption of chemical pesticides and their environmental impact on farms [13,19,20,21,22]. For this purpose, some researchers [23,24] used remote sensing technologies to discriminate weeds, crops, and soil based on reflectance measurements at different wavelengths, while others [25,26,27,28,29,30] developed image processing systems for plant classification-based weed detection on the basis of color, texture, and morphological features. Machine vision based on image processing has been applied in two data collection forms of two-dimensional (2D) vision and 3D (stereo) vision. Machine vision systems based on two-dimensional (2D) image processing have some shortcomings when using 2D cameras. First, variations in outdoor illumination affect the quality of images capture by 2D cameras; therefore, it is needed to cover the camera’s field. Second, the overlap of different plant parts together can cause difficulty in diagnosis of weeds from crops. To discriminate between crops and weeds, pattern recognition methods and algorithms (e.g., statistical pattern recognition, a support vector machine (SVM), artificial neural networks (ANNs), decision trees, fuzzy logic, etc.) were applied in 2D vision processing. 

Zheng et al. (2017) developed and tested a new classification method based on color indices and support vector data description (SVDD) [5]. Results of a 3-year case study showed an overall accuracy of 90.19%, 92.36%, and 93.8% in the first, second, and third years, respectively. 

Sujaritha et al. (2017) proposed a fuzzy real-time classification technique for extracting leaf texture. The results of this research showed that the overall accuracy of this system for detecting weeds was 92.9% [29]. 

Sabzi and Abbaspour-Gilandeh (2018) presented an expert computer vision system for the detection of potato plants from weeds. They recorded videos from a 4 ha potato field located in Kermanshah, Iran, using a camera operated at 0.13 m/s under natural light conditions. They proposed two optimized neural network metaheuristic algorithms to select the most effective features and then classify the plants. Accordingly, five effective features were selected from the original pool of 186 features by using a hybrid neural network-cultural algorithm method. Next, the neural network was combined with a harmony research algorithm to detect weeds from potato plants with 98.38% accuracy in a processing time of 0.8 s per frame [26]. 

Nguyen et al. (2013) used genetic programing for discrimination of rice and other leaf classes. In order to evaluate the classifier, they further used a scanning window of 20 × 20 pixels on a test image and applied the classifier on every single pixel of the window based on a color threshold, achieving an accuracy of 90% [31]. 

Partel et al. (2019) designed and developed a smart sprayer using machine vision and artificial intelligence to discriminate weed and non-weed objects. For precise spraying, this targeted system was integrated with a novel precision spraying system equipped with a state-of-the-art weed detection system and a weed mapping system. The results showed that the application of this system reduced the required quantity of agrochemicals compared to the traditional broadcast spraying systems that usually use the entire field [32]. 

Bakhshipour and Jafari (2018) tried to use a SVM and ANN to detect weeds across a sugar beet field. For each type of weed/crop, they built a pattern from several shape features and used this pattern for classification. This study showed higher classification accuracy of the SVM (up to 93.39%) compared to the ANN (up to 92.67%) [33]. 

Hamuda et al. (2017) used the HSV color space and morphological operations for automatic discrimination of cauliflowers, weeds, and soil in neutral condition. In the proposed algorithm, a target region was found by filtering each of the three HSV (Hue, Saturation, and Value) channels between the minimum and maximum thresholds before applying morphological operations onto the selected region. Finally, the statistical moment method was applied on the video frames to determine the position and mass of the object. For evaluating the algorithm, the result was compared with ground truth methods, returning a classification sensitivity of 98.91% and a precision of 99.04% [34]. 

Two cameras are used in passive stereo vision techniques. A baseline separates these two cameras and they capture two (left and right) images in the same scene and at the same time. For getting disparity maps for depth calculation, this technique depends on correspondence matching between left and right images. Correspondence matching between left and right images not only uses computation intensively in real-time, but also adequate textural information is needed to guarantee the reliability and accuracy of this correspondence matching. Active stereo vision, compared with passive stereo vision approach, uses a structured light pattern (grid, lines), which is projected onto the surface of a detected object to reconstruct its 3D shape. This technique may take an unacceptable processing time for in-field applications [35,36,37].

Jeon et al. (2011) developed a machine vision system by using a stereo camera for discriminating crop plants, weeds, and soil on the images taken from fields under natural illumination conditions in the early growth stage of the plant. The proposed algorithm included the normalized excessive green modification, statistical threshold estimation, image segmentation, median filter application, morphological feature extraction, and ANN. Results of this study showed that the ANN could detect the crop plants correctly with an accuracy of up to 95.1% [38]. 

Tilneac et al. (2012) developed a 3D stereo vision by using two web cameras to discriminate weeds and plants in laboratory conditions. For this propose, they used green color and depth information. The result showed the feasibility of such discrimination only when there was a significant height difference between the crop and the weed [39].

The main objective of this study was to develop a new utilization of the stereo vision system for discrimination of the rice plant and two types of weed groups by using ANNs and two metaheuristic algorithms for feature selection and classification. Unlike the common methods of using stereo vision that utilize point clouds and disparity maps for weeds and crop detection by their phenotypes and height, the innovation of the presented research lays in the new application of a stereo camera for rice and weeds identification by splitting the left and right channels of the stereo recorded video. Classification results of two 2D extracted videos were then compared to obtain high-accuracy plant classification and, hence, smart weed-control under natural-illumination under in-field conditions. Little research on weed detection of rice fields using image processing due to the specific conditions of rice field has led to the use of a combination of 2D and 3D vision processing advantages to identify weeds of a rice field. In principle, there should not be much difference between images taken from one scene by two similar lenses located close, but in practice, due to overlapping and shadows caused by vegetation density, there is some ambiguity that can be eliminated by using multiple source information (cameras) and integrating their outputs.

## 2. Materials and Methods

### 2.1. Plant Material

In this study, a rice cultivar (*Tarom Mahali*) and two common types of weeds (narrow-leaf weeds (*Echinochloa crus-galli*, *Paspalum distichum*, and *Cyperus difformis*) and wide-leaf weeds (*Alisma plantago-aquatica* and *Eclipta prostrata)*) were focused on because of their abundance in the selected region. Another reason for choosing these species of weeds was their competition with rice in every stage of growth. The study was performed on a 5 ha rice field in Mazandaran, Iran (36°37′48.71″ N, 52°30′11.39″ E), during 2017. The predominant method of rice cultivation in this area is traditional transplanting, which begins around the middle of April. In water and foliar spray are two modes of herbicide application in rice fields. The water method, in addition to the high consumption of herbicides, causes a lot of environmental pollution, for obvious reasons. On the other hand, the foliar spray method in rice fields is a time-consuming activity and requires great care in practice and precision spraying, as a site–specific weed management can increase the efficiency of this method.

### 2.2. Video Data Acquisition 

**Figure 1:**
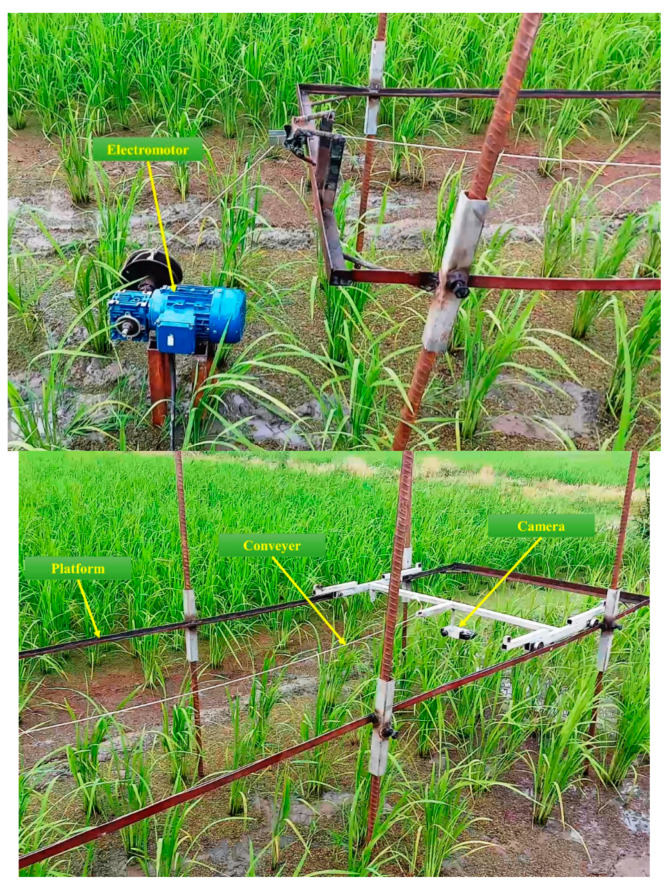
A rail platform for holding the camera and moving it across the field: 3 m length. Note: The growth stage of the rice on the BBCH scale was leaf development and tillering (from 1 week after transplanting to the sixth week) and weeds were leaf development (after three leaves unfolded). Water depth was 10 cm.

The required data was collected in the form of stereo videos by a stereo camera with different channels of each frame extracted. For this purpose, a Fujifilm FinePix Real 3D-W3 digital camera (equipped with a 10-megapixel CCD sensor capturing stereo videos in AVI format (NTSC)) with a sensitivity of iso 400 and frame resolution of 640 × 480 pixels (30 fps) was used. For holding the camera and moving it across the field, a rail platform was designed (Figure 1). The camera was attached to the conveyor at a height of 70 cm from soil surface (30 cm from the tip of the top most leaf, approximately) and moved along a 3 m length rail route at 0.10 m/s during the video acquisition in the rice field. An inverter-controlled electromotor was used to move the conveyor. Data analysis was performed using MatLab software 2018 on a computer system with an Intel Core i5-2540m 2.6 GHz CPU and 4 GB of RAM running a 64-bit operating system. Videos were recorded of the rice plant from 2–3 weeks old after transplanting (rice growth stage from code 14 to code 25 on the BBCH scale) [40], at which time the herbicide application would minimize weed competition. An attempt was made to collect as much data as possible in cloudy conditions, with 850–1200 lux illumination.

### 2.3. Pre-Processing and Segmentation

**Figure 2:**
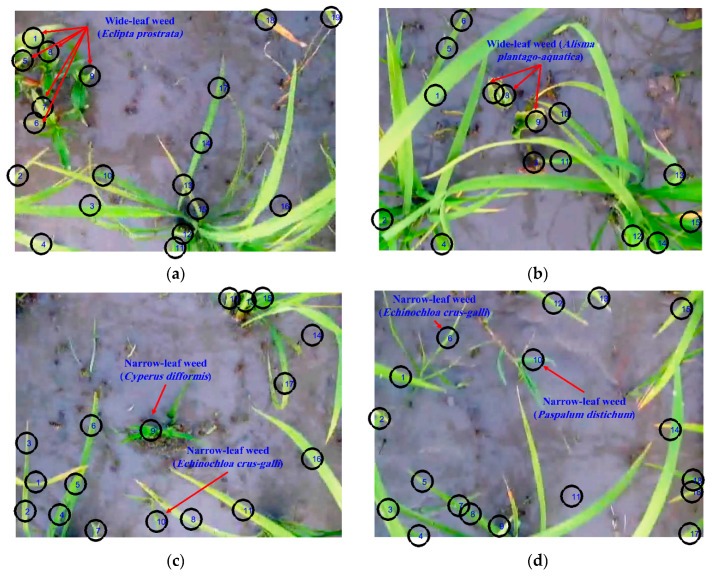
Segmentation of the green components (weeds and rice crop) of sample frames for (**a**) wide-leaf weed (*Eclipta prostrata*), (**b**) wide-leaf weed (*Alisma plantago-aquatica*), (**c**) narrow-leaf weeds (*Cyperus difformis* and *Echinochloa crus-galli*), and (**d**) narrow-leaf weeds (*Echinochloa crus-galli* and *Paspalum distichum*).

To achieve the aim of this research, the captured stereo video was decomposed into two 2D video channels (left and right) by programming in the FFmpeg software. The videos were then converted to their constituent frames by a code written in Matlab software. In order to remove unneeded information from the frames, segmentation was performed to separate different regions of the frames according to common features, thereby discriminating the plants (either weeds or the crop) and the background (soil, rocks, and residue). Considering the accuracy and fatness of the color-based segmentation, different color spaces (e.g., RGB, HIS, HSV, YIQ, YCbCr, and CMY) were surveyed for selecting the most efficient color model for segmentation. The best results were obtained with RGB color space because of the presence of the green objects on the frames. Equation (1) identifies a pixel as a plant if its green component (*II*(*:,:,*2)) is dominant over its blue (*II*(:,:,3)) and red (*II*(:,:,1)) components. Numerous studies have been conducted to segment green plants from the background using RGB indices [4,41]. Considering the camera motion speed (0.10 m/s) and natural condition effects, the optimal threshold of 140 for green channels of RGB color space was adopted (in the range of 20–250), according to the method described in Reference [42], by checking different images for eliminating incomplete green components versus the required processing time. Figure 2 shows sample frames on which the green components were segmented. For shape feature extraction, the result of this segmentation had to be converted to binary images, a process that is always associated with the generation of unwanted noise and holes on the image. For solving this problem, morphological closing operation was employed, connecting thin broken components and filling the small holes [43]. A combination of two dilation and erosion operations as a closing filter was used to soften the contours of the object.

(1) mask = II(:,:,1)< II(:,:,2) & II(:,:,3)< II(:,:,2) & II (:,:,2) ≥ 140

If G(x, y) ≥ 140, then pixel (x, y) is considered as the object (foreground), else (x, y) is considered as the background, since (x, y) is the position of the pixel under consideration, which in turn depends on both the sample image and the capture system used. A series of algorithms were used to extract information from images and process them. In this context, masks were filters used to remove unwanted information and image noise.

### 2.4. Feature Extraction

As a common operation in machine learning, feature extraction has been studied by many researchers [4,26,27]. In principle, feature extraction is performed based on the measurement of geometric properties (such as size and shape) and surface characteristics (such as color and texture) of different objects across an image. In this research, in order to achieve accurate identification of the rice and weeds, a total of 302 color, shape, and texture features were extracted. The texture has been frequently used to classify and discriminate crops and weeds, where leaf occlusion and overlapping are problematic [44]. Two texture-based feature extraction methods have been utilized in the relevant literature; these have been based on either the gray level co-occurrence matrix or histogram analysis. The former technique considers the gray scale values of the pixels and seeks to capture repetitive patterns in an image [45]. The co-occurrence matrix C (i, j) computes the co-occurrence of pixels with gray values i and j at a distance d, defined as a length in a polar coordinates system along an orientation declared by θ. In this study, 146 texture features were extracted at four different neighborhood angles (θ = 0°, 45°, 90°, and 135°) and at a unit distance of 1. The histogram analysis was performed to extract two texture features. Many researchers have used the color characteristics for feature extraction as a key step of image processing [5,30]. Accordingly, 127 color features (based on average and standard deviation of pixel values in either of the three channels of six color spaces (RGB, HIS, HSV, YIQ, CMY, and YCbCr) and vegetation indices [46] were extracted in this study. As a visional feature, the shape can be used for feature extraction accurately when no occlusion or overlapping of the leaves exists [47,48]. In this study, 29 shape features were extracted for each object in this study.

### 2.5. Effective Feature Selection 

Not all of the features extracted from an image are equally informative. Indeed, some of the features may be noisy, correlated to other features, or even irrelevant. In order to save the processing time for pattern recognition and increase the classification accuracy, it is very important to select the most effective features among the entire pool of extracted features. For this purpose, several authors have used statistical techniques, as well as ANN-based methods [18,49]. An ANN is a network of artificial neurons connected together to mimic the function of a human’s brain and is designed to perform a non-linear signal processing, classification, or regression. Thanks to its capabilities for learning complex functions (supervised learning from known desired output input samples), an ANN can be used to process parallel information (e.g., identification of crop plants on field images) [50,51], and, once it has properly learnt from examples, to generalize to un-seen input data. The present work proposes a hybrid artificial neural network–particle swarm optimization (ANN-PSO) algorithm to help the search, process, and choose the most relevant features from the entire pool of extracted features. The PSO is a bio-inspired computational algorithm that works on the basis of randomly selected populations, called particle swarms. The particles move collectively within a search space in order to achieve the optimal solution, resembling so-called ‘bird behavior’. The movement of the particles in the search space is managed by their velocities. An objective function is utilized to evaluate a fitness value for each particle in the PSO. The velocity and position of particles are iteratively updated until the goal is met. To select the most significant features, among the 302 features extracted by ANN-PSO, total input dataset samples (objects) extracted from frames were split into either a training subset (70%) a validation subset (15%), or a test subset (15%), following a uniform random selection of input samples. The PSO algorithm was used to form subsets of different sizes and send them to a multi-layer perception (MLP) neural network. Table 1 shows the empirically tuned parameters of the MLP and PSO.

### 2.6. Classification

The last step of the application of computer vision for weed and rice detection is the classification; the process of analyzing various properties of the image features and clustering them into various predefined classes [52].

Different classifiers were used for image classification, such decision trees, ANNs, and SVMs. Classification accuracy is directly associated with the choice of the classifier. Of the mentioned classifiers, an ANN was utilized in this study because of its following advantages:

- High computation speed;
- Ability to efficiently handle noisy inputs;
- Data-driven nature, thanks to learning from the training data.

An ANN is formed of successive layers, with each layer being composed of a set of neurons. Weighted connections link the neurons of each layer to all neurons of the preceding and proceeding layers. The first layer (input layer) receives inputs by interacting with the environment, and the last layer (output layer) provides the processed data. The artificial intelligence (AI) incorporated into the proposed classifier in this research was a MLP neural network—a technique inspired by the biological network of neurons. This classifier included a series of parameters (number of neurons in each layer, transfer functions, back-propagation weighting factors and bias learning function, and back-propagation training function), which together defined the network structure. For optimizing the network parameters and successfully executing the ANN, the bee algorithm (BA), with multiple iterations, was used in this study. The BA is an optimization algorithm inspired by the honey bee’s foraging behavior [53], where a group of discoverer bees are randomly flown to the field from one patch to another. After returning to the hive, they start dancing [54]. This dance transfers some information about the flower patch, such as the direction toward, the distance to, and the quality of the found flowers [55]. This information helps other bees watching the dance find the flower with no more guidance. In this way, the colony gathers enough food quickly. For performance evaluation of the proposed ANN-BA, the K-nearest neighbors (KNN) classifier was used as a reference to compare the classification results. The KNN is a supervised machine-learning algorithm for statistical classification. It is a popular, simple, and easy-to-use classifier [56] that takes the training data in the training stage and classifies the testing data by comparing them to the training data. In order to evaluate the capabilities of the proposed classifiers for predicting respective classes, 70% of the input data was selected for training and validation of the network, with the remaining 30% of the data used for testing the accuracy of the classifier. 

### 2.7. Proposed System for the Classification of Rice and Weed Plants Inside Rice Fields

**Figure 3:**
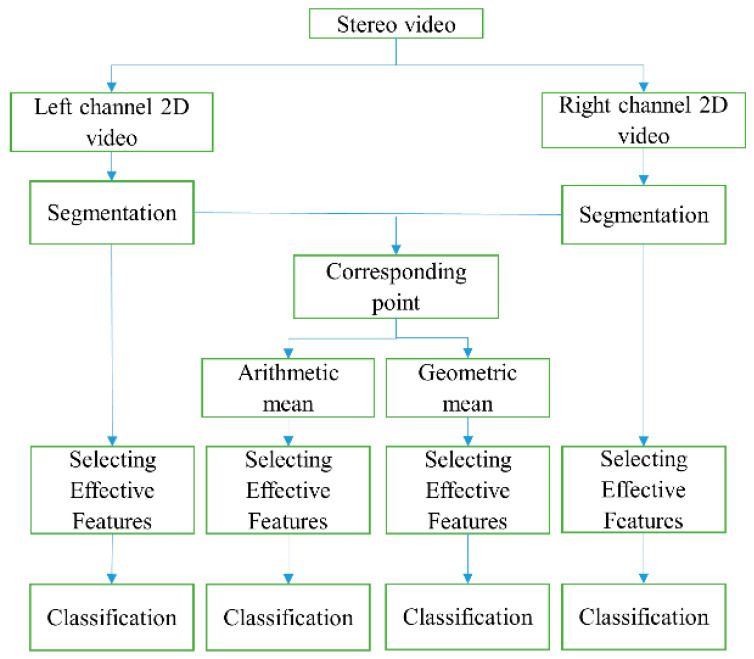
Flowchart of the proposed system for the classification of rice and weed plants inside rice fields by recording stereo video and decomposing the video into right and left channel data.

In order to achieve high-accuracy discrimination of rice and weeds based on stereo vision under natural illumination, four categories of data were processed and classified. In addition to classification of the left and right channel data separately, arithmetic and geometric means of the corresponding features on the two channels were calculated and classified. Finally, classification results of right channel and left channel data and the arithmetic and geometric mean were compared to select the best classification scheme. For this purpose, the pre-processing, segmentation, effective feature extraction, and classification steps, as described in Figure 3, were performed for all four data sets. Figure 3 presents the complete flowchart of the proposed system for rice and weeds classification.

### 2.8. Arithmetic and Geometric Means

Arithmetic and geometric means are two commonly used mathematical terms differing in the method of calculation. Arithmetic mean (or simply the mean) is calculated by adding up all the numbers in the dataset and dividing the result by the total number of data points, while the geometric mean is calculated by multiplying the numbers in the dataset and taking the nth root of the result, where n is the total number of data points [57]. In this study, after extracting the features of the left and right channels and finding the corresponding points, the arithmetic and geometric mean of the points were calculated according to Equations (2) and (3).

(2) $$\mathrm{Arithmetic}\;\mathrm{mean}=\frac{\mathrm{Right}\;\mathrm{channel}\;+\;\mathrm{Left}\;\mathrm{channel}}{2}$$

(3) $$\mathrm{Geometric}\;\mathrm{mean}=\sqrt{\mathrm{Right}\;\mathrm{channel}\;\times \;\mathrm{Left}\;\mathrm{channel}}$$

## 3. Results and Discussion

### 3.1. Effective Feature Extraction with ANN-PSO

The results of effective feature extraction by the hybrid ANN-PSO algorithm for the two channels and arithmetic and geometric means are shown in Table 2. As shown in this table, 6 features were selected from the pool of 302 extracted features and most of the selected features were either color or texture features. Table 3 presents the definitions of the selected features in the four categories.

### 3.2. Classification Using Hybrid Metaheuristic Algorithms

As mentioned earlier, the aim of using the BA was to optimize the parameters of the MLP neural network. For this reason, the training process was iterated for 1000 cycles for all of the four categories. Specifications of the used MLP and optimal values of its parameters are shown in Table 4.

#### 3.2.1. Classification Using Hybrid ANN-BA

The result of performing the hybrid ANN-BA for classification of the test set for the left channel, right channel, arithmetic mean, and geometric mean to three classes (Class 1: rice, Class 2: narrow-leaf weeds, and Class 3: wide-leaf) are tabulated, in the form of the best confusion matrix and overall accuracy, in Table 5. Confusion matrix indicates the performance of the classification model, which is typically a learning-supervised model on the testing dataset by comparing predicted classes (Column) against the actual ones (Row). The results show that the proposed classifier could correctly classify the rice in all four categories. The misclassification cases were much more in narrow-leaf weeds class rather than the other classes, due to the close similarity between the first and second classes. Misclassification of the wide-leaf weeds class under the first (rice) or second classes (narrow-leaf weeds) and vice versa could be a result of moving the camera in the field and recording the video under natural light conditions (Video S1 and Video S2). As shown in the tables, the overall accuracy of the ANN-BA classifier for the right and left channel data (Video S3) achieved 88.74% and 87.96%, respectively. Taking the arithmetic and geometric means as a basis, the accuracy of 92.02% and 90.7% were obtained, respectively. In fact, the classification accuracy increased when the process was based on the arithmetic and geometric means of the corresponding points from the right and left channels. This result expresses the inadequacy of a single 2D movie recorder when classification under natural field condition was concerned. On the other hand, higher classification accuracy was obtained with the arithmetic mean (Video S4) rather than the geometric mean (Video S5), because the geometric averaging was applicable to only positive values, always returning a lower value than the arithmetic mean, while the arithmetic mean covered both positive and negative values, making its value always greater than the geometric mean. This finding further indicates that there were not any outliers in the dataset.

#### 3.2.2. Classification Using KNN Classifier

In order to investigate the performance of the proposed ANN-BA classifier, the KNN classifier was used as a reference for comparison. The results of applying the KNN classifier on the test set for the left channel, right channel, arithmetic mean, and geometric mean to three output classes of 1-rice, 2-narrow-leaf weeds, and 3-wide leaf weeds are presented in Table 6. The result shows that the KNN classifier could classify the first and third classes more correctly than the second class, just like the proposed ANN-BA classifier. Indeed, misclassification of rice under the second class was the most frequent problem, confirming the similarity of rice and narrow-leaf weeds, as explained previously. In total, the KNN generated more cases of misclassification under all of the three classes, as compared to the proposed ANN-BA classifier, returning classification accuracies of 76.62%, 85.59%, 85.84%, and 84.07% based on the right and left channel data and arithmetic and geometric means, respectively. This comparison highlighted the larger capability of the machine-learning classification method compared to the statistical method. Statistical methods are merely based on mathematical equations and some background assumptions, while machine-learning work, by learning from training examples, resembles human cognition.

To check for reliability of the proposed classifier, the training process was iterated for 1000 cycles, followed by calculating the mean and standard deviation (STD) values of accuracy. Table 7 reports the results for the proposed hybrid ANN-BA and the KNN classifier in each of the four categories. The results indicated low STD values for both classifiers under three classes in the four categories, proving proper training of the classifiers. The higher mean value of accuracy for the proposed hybrid ANN-BA, as compared to the KNN classifier, indicated better training performance of the ANN-BA classifier.

#### 3.2.3. Classification Performance Evaluation by receiver operating characteristic (ROC) Curves

Performance evaluation is an essential task in machine learning. The receiver operating characteristic (ROC) curve is a graphic representation that shows the performance of a classifier over all possible thresholds. The Y-axis and X-axis of an ROC curve represent the true positive rate (TPR) and false positive rate (FPR). Since the TPR and FPR are defined as “sensitivity” and “1-specificity”, respectively, the ROC curve is sometimes introduced as the “sensitivity vs. 1-specificity” plot. Each prediction result of the confusion matrix represents a point on the ROC curve. In the ROC domain, the point (0, 1) represents the perfect classification. The points from a random guess lie on the diagonal line that divides the ROC domain from the left bottom to the top right corner. The points above the diagonal show good (or better than random) classification results, while those below the line show poor (worse than random) results. In order to evaluate the classifier performance more accurately, in addition to the ROC curve, the ROC-best curve was studied in this work.

**Figure 4:**
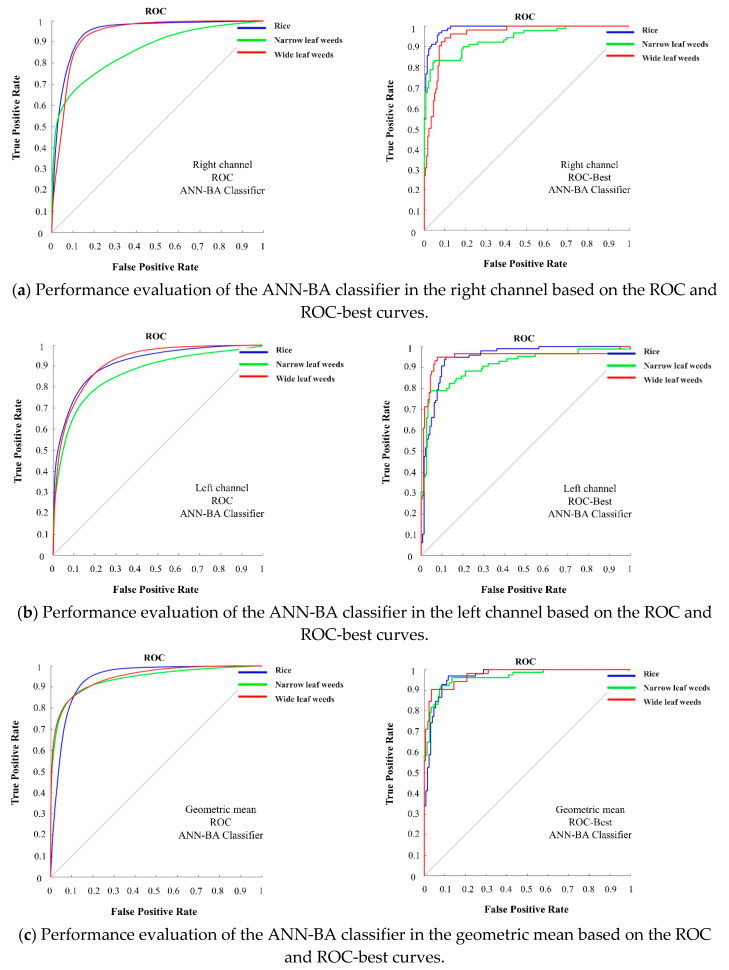

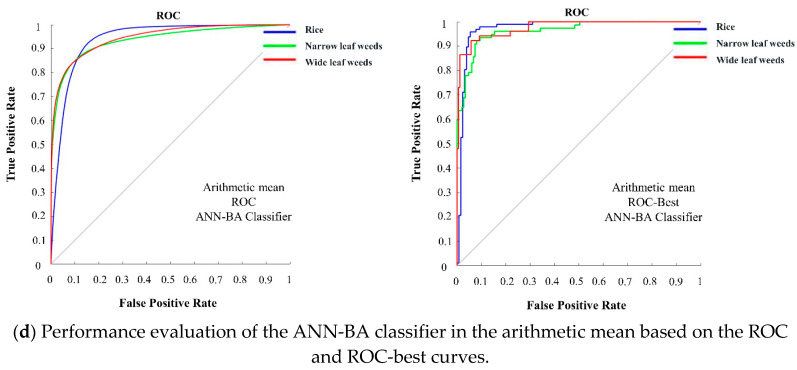
Performance evaluation of the ANN-BA classifier on the test dataset in the four categories (right, left, geometric mean, and arithmetic mean) based on the ROC and ROC-best curves related to the three classes (rice, narrow-leaf weed, and wide-leaf weed).

Figure 4 shows the classification performance of the two classifiers on the test dataset from the right and left channel cameras and arithmetic and geometric means in the form of the ROC and ROC-best curves. Based on this figure, the ANN-BA classifier outperformed the KNN classifier in all of the four categories. Moreover, both of the classifiers returned better results based on the arithmetic and geometric means data rather than either right or left channel data alone. To prove this, the area under the curves were calculated (Table 7), showing a larger value for the proposed hybrid ANN-BA classifier compared to the KNN classifier under all of the three classes in all of the four categories. Given the novelty of the proposed methodology, unfortunately, we were not able to find similar approaches to directly compare results with; thus, we had to compare with others. Cheng and Matson (2015) used feature-based methods for rice and weed identification. In this research, the Harris corner detection algorithm was applied to find characteristic points, such as the tips and ears of the leaves. Multiple features were extracted for each point and fed into three machine-learning algorithms (decision tree, SVM, and neural network) to distinguish between weeds and rice. The decision tree classifier returned an accuracy of 98.2%, while the corresponding figures to SVM and naive Bayes were 95.3% and 93.1%, respectively. They used a clustering algorithm for noise removal and for clustering the most similar objects into three clusters. Working on presumably edited/processed images retrieved from the Internet, rather than taking actual photos, they ended up with artificially high accuracies. Sabzi et al. (2018a) classified potato plants among three different kinds of weeds by a novel computer vision system. They used 2D video images acquired under outdoor lighting conditions and used them to train and test two metaheuristic algorithms (ANN-CA for selecting effective features and ANN-HS for classification) for optimizing the performance of a neural network classifier. They further compared their results against the KNN, a statistical classifier. The result of this study showed that the proposed expert system could achieve a high accuracy of 98.38%, as compared to the 88.25% accuracy achieved by the KNN classifier [27]. In another study by Sabzi et al. (2018b), a digital camera was used as a video acquisition system for classification of potato plants among three weed types using three hybrid ANN classifiers based on an ant colony algorithm, radial basis function ANN, and discriminant analysis, leading to ultimate accuracies of 98.13%, 91.23%, and 70.8%, respectively [58]. A comparison between the results of this research and the proposed methodology in the present study revealed the capability of the proposed hybrid ANN-BA classification algorithm. The mentioned studies have been done in the dry fields with low density of weeds and crops, while the present study focused on a densely cultivated rice field with special conditions (moving the video capturing device on wet soil), where high accuracy could not be expected with a single digital camera (either right or left channel). This emphasizes the efficiency of using a pair of cameras (stereo vision) for increasing the classification accuracy.

The average time for image acquisition and pre-processing operations was 0.233 s. Altogether, the feature extraction stage needed 0.175 s and the classification time was 0.164 s on average, thus resulting in an average total time of 0.633 s per frame.

Considering that the identification of weeds from the rice crop was the first stage of site-specific weed management to control weeds, the results of this study showed that the proposed methodology with the hybrid ANN-BA classification algorithm had a good performance in discriminating rice and weeds (Table 8 and Table 9).

**Figure 5:**
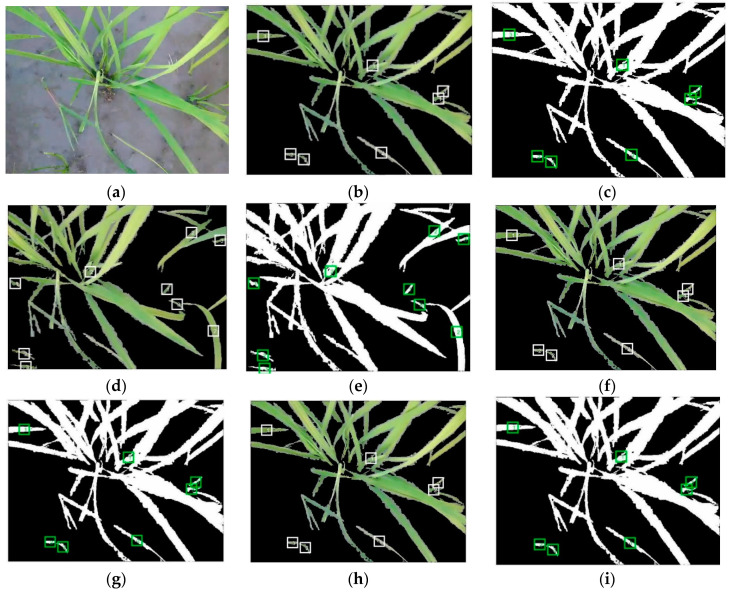
Segmentation and classification of rice and weeds in color and binary images for a frame. (**a**) Original frame, (**b**) color model of left channel, (**c**) binary model of left channel, (**d**) color model of right channel, (**e**) binary model of right channel, (**f**) color model of arithmetic mean, (**g**) binary model of arithmetic mean, (**h**) color model of geometric mean, and (**i**) binary model of geometric mean.

Finally, Figure 5 contains results of the proposed computer vision expert system in segmentation and classification of rice and weeds in the four categories for a frame. It can be observed that the system was capable of more carefully detecting and classifying all existing plants in arithmetic mean (Video S6) and geometric mean (Video S7) categories than the left channel (Video S8) and right channel (Video S9) categories.

## 4. Conclusions

Application of a new stereo vision-based method for weeds and crop classification across a densely cultivated rice crop was developed. For this aim, stereo videos were recorded in the rice field and decomposed into right and left channel data. All weeds in the rice field were classified under narrow-leaf weeds and wide-leaf weeds. In order to enhance the classification accuracy, two metaheuristic algorithms, namely PSO and BA, were used to optimize the performance of the neural network for selecting the most effective features and classification, respectively. Results of this classification method were compared to those of the KNN classifier on a set of test data consisting of right channel and left channel data and arithmetic and geometric means of the corresponding points on the two channels. Results proved the promising capability of the stereo vision technology proposed, averaging the corresponding points on different channels and the proposed hybrid ANN-BA classifier for increased classification accuracy. Future research work may evaluate the proposed methodology over other varieties of rice and on different density cultivated crops, under different farm conditions.

## Figures and Tables

**Table 1:** The parameters of the multi-layer perception (MLP) and particle swarm optimization (PSO) for the hybrid artificial neural network (ANN)-PSO for selecting the most significant features.

MLP Parameters	PSO Parameters
One input layer	Swarm size: 30
One hidden layer with 10 neurons	Maximum iteration: 20
One output layer with 3 outputs.	Inertia weight damping ratio: 1
Classic Levenberg–Marquardt training function	Maximum variation size: 1
	Minimum variation size: 0
	Inertia rate: 1
	Velocity Maximum value:
	0.1×(VarMax-VarMin)
	Velocity minimum value: -VelMax

**Table 2:** The most effective features selected by the proposed hybrid ANN-PSO algorithm from the left and right channel data and arithmetic and geometric means.

Category	Selected Effective Features					
Left channel	EXY-YIQ	Elongation Feature	Cluster Prominence-45	Rn	Inverse Difference-45	Entropy-45
Right channel	Convexity	ExG-RGB	CIVE-HSV	Cluster shade-90	CIVE-RGB	Difference entropy-0
Arithmetic mean	Sum entropy-0	Information measure of correlation-0	CIVE-RGB	Autocollelation-90	Coefficient of variation--90	WL
Geometric mean	Inverse difference normalized-135	WL	CMP	Std-Cb	Entropy	ExM-CMYYY

**Table 3:** Formal definition of selected features inside the four categories under consideration: description and feature name.

Description	Selected Feature Name
Excess yellow from YIQ color space	EXY-YIQ
Elongation feature = (L − W)/(L + W) L = length and W = width	Elongation feature
*Clumster prominence* = Σ*_i_*Σ*_j_*(*i* + *j* − *μ_i_* − *μ_j_*)^4^*N_g_(i,j)* *N_g_* = $\frac{g\left(i,j\right)}{\sum i\sum j\;g\left(i,j\right)}$ *(The normalized co-occurrence matrix)*	Cluster prominence
Rn = R/(R + G + B), (The normalized first component of RGB)	Rn
Inverse Difference = $\overset{n-1}{\underset{i=0}{\sum }}.\overset{n-1}{\underset{j=0}{\sum }}\frac{Ng\left(i,j\right)}{1+\left[i-1\right]}$	Inverse Difference
*Entropy* = −ΣΣ*N_g_(i,j)log_2_ N_g_(i,j)*	*Entropy*
A measure of the curvature	Convexity
ExG-RGB = 2 × Gn − Rn − Bn, (Excess green)	ExG-RGB
Color index for extracted vegetation cover in HSV color space	CIVE-HSV
cluster Shade = ΣΣ*(i + j − μ_i_ − μ_j_)*^3^*N_g_(i,j)*	*cluster Shade*
CIVE-RGB = 0.441 × Rn − 0.811 × Gn + 0.385 × Bn + 18.78 (Color index for extracted vegetation cover)	CIVE-RGB
Difference entropy = −Σ*p_x__−__y_(i)* ln [*p_x__−__y_(i)*], *p_x__-__y_(k)* = $\underset{i,j:\left[i-j\right]=k}{\sum }Ng\left(i,j\right)\;for\;k=0,\ldots ,Ng-1$	Difference entropy
*Sum Entropy* = −Σ*p_x__+__y_(i)*log(*p_x;__+__y_(i))* *p_x__+__y_(k)* = $\underset{i,j:i+j=k}{\sum }Ng\left(i,j\right)\;for\;k=2,3,\ldots ,2l$	*Sum Entropy*
IMC = $\frac{ENT-HXY1}{max\left(Hx,Hy\right)}$ *HXY1* = $-\overset{n-1}{\underset{i=0}{\sum }}\overset{n-1}{\underset{j=0}{\sum }}Ng\left(i,j\right)\ln\left[Nx\left(i\right).Ny\left(j\right)\right],$ *N_x_(i)* = $\overset{n-1}{\underset{i=0}{\sum }}Ng\left(i,j\right)$, *N_y_(i)* = $\overset{n-1}{\underset{j=0}{\sum }}Ng\left(i,j\right)’$, *H_X_*: *Entropy of N_x_ and H_y_: Entropy of N_y_*	Information measure of correlation
*Autocorrelation* = *ΣΣ(ij)N_g_(i,j)*	*Autocorrelation*
Standard deviation to mean of co-occurrence matrix	Coefficient of variation
WL = Width/Length	WL
IDN = $\overset{n-1}{\underset{i=0}{\sum }}.\overset{n-1}{\underset{j=0}{\sum }}\frac{Ng\left(i,j\right)}{1+\frac{{\left[i-1\right]}^{2}}{L^{2}}}$	Inverse difference normalized
CMP = $\frac{p^{2}}{4\pi A}$ (Compression) A:area, p:perimeter	CMP
Standard deviation of Cb from YCbCr color space	Std-Cb
Excess magenta From CMY color space	ExM-CMYYY

**Table 4:** The optimized parameters for classification using the hybrid artificial neural network bee algorithm (ANN-BA).

Number of Hidden Layers	Number of Neurons	Transfer Function	Back Propagation Network Training Function	Back Propagation Weight/Bias Learning Function
2	First layer: 20 Second layer: 12	First layer: tansig Second layer: satlins	trainrp	learngd

**Table 5:** Confusion matrices and accuracy of the ANN-BA classifier for the left channel, right channel, arithmetic mean, and geometric mean (test set).

Left channel	Rice	Narrow-leaf weeds	Wide-leaf weeds
Rice	89	6	2
Narrow-leaf weeds	12	67	6
Wide-leaf weeds	2	1	56
Accuracy = 87.96%			
**Right channel**	**Rice**	**Narrow-leaf weeds**	**Wide-leaf weeds**
Rice	86	3	1
Narrow-leaf weeds	6	73	10
Wide-leaf weeds	2	4	46
Accuracy = 88.74%			
**Arithmetic mean**	**Rice**	**Narrow-leaf weeds**	**Wide-leaf weeds**
Rice	91	5	1
Narrow-leaf weeds	6	69	2
Wide-leaf weeds	1	3	48
Accuracy = 92.02%			
**Geometric mean**	**Rice**	**Narrow-leaf weeds**	**Wide-leaf weeds**
Rice	91	6	0
Narrow-leaf weeds	7	67	3
Wide-leaf weeds	3	2	47
Accuracy = 90.70%			

**Table 6:** Confusion matrices and accuracy of the K-nearest neighbors (KNN) classifier for the left channel, right channel, arithmetic mean, and geometric mean (test set).

Left channel	Rice	Narrow-leaf weeds	Wide-leaf weeds
Rice	83	8	6
Narrow-leaf weeds	10	65	10
Wide-leaf weeds	0	0	59
Accuracy = 85.89%			
**Right channel**	**Rice**	**Narrow-leaf weeds**	**Wide-leaf weeds**
Rice	65	19	6
Narrow-leaf weeds	17	60	12
Wide-leaf weeds	0	0	52
Accuracy = 76.62%			
**Arithmetic mean**	**Rice**	**Narrow-leaf weeds**	**Wide-leaf weeds**
Rice	83	8	6
Narrow-leaf weeds	11	62	4
Wide-leaf weeds	3	0	49
Accuracy = 85.84%			
**Geometric mean**	Rice	**Narrow-leaf weeds**	**Wide-leaf weeds**
Rice	78	15	4
Narrow-leaf weeds	12	60	5
Wide-leaf weeds	0	0	52
Accuracy = 84.07%			

**Table 7:** Mean and standard deviation (STD) values of accuracy for the proposed hybrid ANN-BA and the KNN classifiers: three classes and four classifier categories.

Right Channel					
**Hybrid ANN-BA**	**Mean**	**STD**	**KNN**	**Mean**	**STD**
Rice	0.9446	0.0212	Rice	0.7224	0.0265
Narrow-leaf weeds	0.8596	0.0314	Narrow-leaf weeds	0.6942	0.0272
Wide-leaf weeds	0.9323	0.0289	Wide-leaf weeds	0.9004	0.0315
**Left Channel**					
**Hybrid ANN-BA**	**Mean**	**STD**	**KNN**	**Mean**	**STD**
Rice	0.9100	0.0275	Rice	0.8256	0.0238
Narrow-leaf weeds	0.8625	0.0275	Narrow-leaf weeds	0.7948	0.0273
Wide-leaf weeds	0.9132	0.0376	Wide-leaf weeds	0.8961	0.0305
**Arithmetic mean**					
**Hybrid ANN-BA**	**Mean**	**STD**	**KNN**	**Mean**	**STD**
Rice	0.9563	0.0165	Rice	0.8091	0.0240
Narrow-leaf weeds	0.9330	0.0179	Narrow-leaf weeds	0.7993	0.0254
Wide-leaf weeds	0.9653	0.0211	Wide-leaf weeds	0.9214	0.0272
**Geometric mean**					
**Hybrid ANN-BA**	**Mean**	**STD**	**KNN**	**Mean**	**STD**
Rice	0.9414	0.0141	Rice	0.7745	0.0254
Narrow-leaf weeds	0.9387	0.0169	Narrow-leaf weeds	0.7625	0.0258
Wide-leaf weeds	0.9478	0.0200	Wide-leaf weeds	0.9493	0.0234

**Table 8:** Mean area under the receiver operating characteristic (ROC) curves (AUC) for the hybrid ANN-BA classifier for rice, narrow-leaf weed, and wide-leaf weed classes: right channel, left channel, arithmetic mean, and geometric mean.

Hybrid ANN-BA	Rice Class	Narrow-Leaf Weeds Class	Wide-Leaf Weeds Class
Right Channel	0.9886	0.9376	0.9561
Left Channel	0.9462	0.9106	0.9483
Arithmetic mean	0.9731	0.9635	0.9765
Geometric mean	0.9668	0.9638	0.9747

**Table 9:** Mean area under the ROC curves (AUC) for the KNN classifier for rice, narrow-leaf weed, and wide-leaf weed classes: right channel, left channel, arithmetic mean, and geometric mean.

KNN	Rice Class	Narrow-Leaf Weeds Class	Wide-Leaf Weeds Class
Right Channel	0.8008	0.7702	0.9497
Left Cannel	0.8931	0.8567	0.9560
Arithmetic mean	0.8793	0.8758	0.9424
Geometric mean	0.8556	0.8393	0.9742

## Supplementary Materials

The following are available online at https://www.mdpi.com/2223-7747/9/5/559/s1, Video S1: Original left channel video of rice field, Video S2: Original right channel video of rice field, Video S3: Rice and weed identification for right channel data, Video S4: Rice and weed identification for arithmetic mean, Video S5: Rice and weed identification for geometric mean values, Video S6: Binary model of arithmetic mean for classification of rice and weeds, Video S7: Binary model of geometric mean for classification of rice and weeds, Video S8: Binary model of left channel for classification of rice and weeds, Video S9: Binary model of right channel for classification of rice and weeds.
